# The Association between Physical Activity, Motor Skills and School Readiness in 4–5-Year-Old Children in the Northeast of England

**DOI:** 10.3390/ijerph182211931

**Published:** 2021-11-13

**Authors:** Dan Jones, Alison Innerd, Emma L. Giles, Liane B. Azevedo

**Affiliations:** 1 School of Health and Life Sciences, Teesside University, Middlesbrough TS1 3BX, UK; a.innerd@tees.ac.uk (A.I.); E.Giles@tees.ac.uk (E.L.G.); 2 School of Human and Health Sciences, University of Huddersfield, Huddersfield HD1 3DH, UK; l.azevedo@hud.ac.uk

**Keywords:** physical activity, motor skills, school readiness, sedentary behaviour

## Abstract

The benefits of being physically active, possessing good motor skills and being school-ready are well documented in early years. Nevertheless, the association between physical activity and motor skills with school readiness remains unknown. Therefore, the aim of this cross-sectional study was to explore the relationship between these variables. We collected data on 326 four to five-year-old children from the northeast of England. Children’s PA (ActiGraph GT1M accelerometers), motor skills (MABC-2 and the locomotor section of the TGMD-2) and school readiness (EYFSP) were measured, and associations between these variables were examined. This study found that, on average, children engaged in more MVPA (99.6 min/day) and less sedentary behaviour (261 min/day) than documented in previous research. Motor-skill scores were consistent with existing literature in early years. A higher percentage of children in the sample (79.6%) achieved school readiness than the average for England. Regression analyses found that motor-skill variables and sedentary behaviour were significantly predictive of school readiness, whereas physical activity was not. Motor skills and sedentary behaviour significantly predict school readiness. Therefore, promoting motor skills and developmentally appropriate sedentary behaviour activities may increase the number of children achieving school readiness.

## 1. Introduction

The transition from preschool to formal schooling is a critical period in young children’s lives. School readiness is described as a child’s readiness to enter formal education, as measured by a range of competency assessments [1]. It is a multidimensional construct that involves cognitive (e.g., listening and attention), emotional (e.g., managing feelings and behaviour), social (e.g., making relationships), physical (e.g., movement and handling) and academic (e.g., mathematics and literacy) competencies that enable children to be prepared to participate in and benefit from formal education [2,3,4]. In addition, this set of competencies may predict future educational outcomes [5] and have a downstream effect later in life on crime, health and mortality [6]. In England, school readiness is assessed by the teacher at reception age when children are 4–5 years old (the year group that directly precedes formal schooling in schools in England), using the early years foundation stage profile (EYFSP) [4]. In England in 2019, 71.8% of children achieved school readiness [7], and inequalities have been demonstrated according to gender, ethnicity, being in receipt of free school meals and special educational needs [6].

School readiness may also be associated with motor skills and physical activity (PA), as these are influencing factors of cognitive skills and academic achievement in the early years [8,9,10,11,12]. Furthermore, motor skills are described within the criteria for a child to be school ready [4]. Given the proposed and demonstrated association between motor skills and PA [13,14,15], it may be possible that increases in PA may lead to an improvement in motor skills, which may result in a child being more likely to achieve school readiness.

Previous research has also demonstrated an association between school readiness and motor skills. Oja and Jurimae (2002) found that motor skills that require concentration and attention were associated with school readiness when assessed using a controlled drawing test [16]. Other studies have also demonstrated an association between motor skills and academic achievement [12,17,18,19], as well as with cognitive abilities [20]. Additionally, gross motor skills have been longitudinally associated with social behaviour [21], self-control, cooperation and a decrease in hyperactivity in young children [22], while fine motor skills have been associated with academic achievement [23,24], attention [25] and executive function [26]. Similarly, intervention studies of preschool children show that school PA promotion [27] and activity breaks [28] can increase educational scores. Findings of a systematic review also revealed that PA had a significant positive influence on language learning and academic achievement in early childhood [10]. Furthermore, UK policy documents state that enhancing PA may increase the likelihood of a child being school-ready [6].

Research in this area is limited, and to the author’s knowledge, no previous research has explored the association between school readiness measured by the UK EYFSP with physical activity and motor skills using objective physical-activity measurement (ActiGraph accelerometer), a standardized assessment of motor skills (Movement Assessment Battery for Children-2 (MABC-2) and the test of gross motor development-2 (TGMD-2). Therefore, the aim of this study was to explore early years PA, motor skills and school readiness levels, as well as to examine whether PA and motor skills predict school readiness.

## 2. Materials and Methods

The study received ethical approval from Teesside University’s School of Health and Social Care Research Ethics Committee (Ref No. 019/18). Recruitment began in September 2018, using convenience and snowball sampling methods. Eighty-six primary schools from a range of socioeconomic areas within the northeast of England were contacted to take part in the study. Schools were asked to disseminate study information to parents of children in reception (children aged 4–5 years); parents who wanted their children to take part were asked to sign a consent form and return it to the school. All children were asked to provide their assent before data collection began. Physical-activity and motor-skills data were collected cross-sectionally over one academic year between October 2018 and July 2019, and school-readiness data were collected in July 2019. Each participant was assigned an ID code to allow the linkage of pseudo-anonymised school-readiness data with the other data collected on the participant.

### 2.1. Demographic and Anthropometric Variables

Parents reported postcode and the child’s date of birth. Depending on the data provided, child’s home or school postcode, with home postcode as preference, was used to determine socioeconomic status (SES) using the index of multiple deprivations (IMD) rank (2019) [29]. Children’s height (to the nearest 0.1 cm) and weight (to the nearest 0.1 kg) were recorded by the main author (DJ) using calibrated digital scales (SECA scales, Hamburg, Germany) and a portable stadiometer (SECA 213, Hamburg, Germany) whilst the children were wearing their school uniform and school shoes. These were used to calculate body mass index (BMI) (kg/m^2^), which was converted to BMI-z using the International Obesity Task Force age and sex-specific BMI cut points [30].

### 2.2. Physical Activity

Children wore a hip-mounted uniaxial GT1M ActiGraph accelerometer (ActiGraph, Pensacola, FL, USA), which has proven validity for this age group [31]. Parents were provided with instructions on how the accelerometer should be worn, and children were instructed to keep the accelerometer on their right hip. Data were recorded in 15 s epochs [32] and processed with ActiLife version 6.5.4 software (ActiGraph, Pensacola, FL, USA). Given the sporadic nature of young children’s movements, non-wear time was defined as 20 min of consecutive zero [33]. The cut points used in the study were derived by Janssen et al. (2013) (sedentary behaviour (SB) = 0–99 counts per minute (cpm), light PA (LPA) = 100–1679 cpm, moderate to vigorous PA (MVPA) = ≥1680 cpm, vigorous PA (VPA) = ≥3370 cpm) [34]. Children were required to wear the accelerometers for eight consecutive days (e.g., Monday–Monday) for all waking hours, except during any water-based activities (e.g., bathing or swimming). Data were only processed for participants with wear times of at least eight hours on any three days [35,36].

### 2.3. Motor Skills

Motor competence was assessed using the MABC-2 [37] and the TGMD-2 locomotor section [38]. All motor-skill assessments took place in school with children wearing a school uniform.

The MABC-2 was selected as a measure of motor skills as it includes an assessment of fine motor skills. Although the primary purpose of the MABC-2 is to identify motor impairments in children aged 3–16, it has been validated [39] and frequently used in early years to measure motor competence [40,41,42,43]. The MABC-2 consists of eight-test items that assess motor-skill proficiency in three domains: manual dexterity (fine motor skills), aiming and catching (object-control skills) and balance.

The raw scores for each task were converted into age-standardised scores, and scores for tasks that required using the dominant and non-dominant hand or leg were averaged. The standardised scores for each task were summed to give a domain score, and each domain was then added to give a total standard score. Children with a standard score of five or less were deemed to have evidence of a motor delay. Children with a standard score of six or seven were deemed at risk of having a motor delay, and children with a standard score of eight or above were deemed to be normally developing. Results of the MABC-2 were presented as total motor skills (total MABC-2 score), fine motor skills (total manual dexterity score in MABC-2), object-control skills (total aiming and catching score in MABC-2) and balance skills (total balance score in MABC-2).

As the MABC-2 does not assess locomotor skills, the locomotor section of the TGMD-2 was used to collect data on locomotor skills such as run, jump, hop, leap, gallop and slide [38]. Each skill, which was performed twice, comprised three to five components. If a child demonstrated a component of the skill, they were given a score of one; if they did not demonstrate the component, they were given a score of zero. The total score for all of the skills were summed to give an overall locomotor- skill score between 0 and 48.

All motor-skill assessments were conducted at school and supervised by a member of school staff. They were conducted by the main author on an individual basis and were scored live. Children wore their school uniform, and tests were conducted in an area suitably sized to allow the assessments to take place, for example, school hall or playground. Occasionally, fine-motor-skill assessments took place in the classroom. Whilst every effort was made to reduce distractions to the participant, this was not always possible.

### 2.4. School Readiness

School-readiness data were provided by the participating schools in an anonymised format in July 2019 using the early years foundation stage profile (EYFSP) [4]. For school readiness the EYFSP assesses five areas of learning, which are divided into twelve early learning goals, as specified in Table 1. Teachers score the child as emerging (1), expected (2) or exceeding (3) for each of the early learning goals in the prime areas of learning, as well as mathematics and literacy, from the specific areas of learning. For a child to be deemed school-ready, they need to achieve a good level of development, which means they must score expected (2) for each of the 12 early learning goals. A child’s school-readiness score (range 12–36) was calculated by totalling the individual scores (1–3) for each early learning goal.

### 2.5. Statistical Analysis

All analyses were conducted using IBM SPSS Statistics (v.26, New York, NY, USA, IBM Corp) software unless otherwise stated. Average time spent in activity and standard deviations (SD) were calculated for SB, LPA, MVPA and VPA, which were reported in min/day, and total PA (TPA), which is reported in counts per minute (cpm). Average score and SD for each motor skill variable (i.e., total motor, fine motor, object control, balance and locomotor skills) was described, as well as the number of children in each motor classification (evidence of a motor delay, at risk of having a motor delay and normally developing). School readiness was reported as the percentage of children who achieved a good level of development and average school-readiness score. Average and SD for age at exposure (child age when PA and motor-skill data were collected), age at outcome (child age when school-readiness data were recorded), BMI-z and SES were also calculated.

Differences between children with and without PA data were calculated using independent *t*-tests for continuous variables and Chi^2^ test for sex and a good level of development. Sex differences were compared for school-readiness score, motor skills, and PA variables using independent *t*-tests, while a Chi^2^ test was used to explore sex differences in a good level of development. We also explored differences in motor skills, PA level, age, BMI-z and SES between children who did and did not achieve a good level of development using independent *t*-tests.

The association between PA, motor skills, age, BMI-z and SES and school-readiness score was calculated using Pearson bivariate correlations. The differences in school-readiness score according to motor-delay classification were examined using one-way ANOVA analyses and post hoc Tukey tests. A Chi^2^ test was used to assess the association between motor-delay classification and achieving or not achieving a good level of development. Logistic regressions were performed to assess the predictive value of MVPA, TPA, SB and all motor-skill variables individually for achieving a good level of development, adjusted for sex, age at exposure, BMI-z and SES as covariates. The odds ratio (OR) of achieving a good level of development, depending on motor-skill classification, was also calculated using the margins command in Stata v.13 software (College Station, TX, USA, Stata Corp). Linear regression analyses were also performed to explore if SB, MVPA, TPA and motor-skill variables individually predicted school-readiness score using the same covariates.

Sensitivity analyses to explore the effect of missing physical-activity data on linear and logistic regression results were conducted using multiply imputed data. Four participants had missing school-readiness data and were therefore excluded from the analysis prior to multiple imputation. Missing data for MVPA, TPA, SB and BMI-z were multiple imputed using the ‘mi impute chained’ command in Stata v.13 software. Outcomes with no missing data (sex, age at exposure, SES, total motor skill, a good level of development and school-readiness score) were used as regular variables in the imputation model. Twenty-five imputed datasets were generated, and Rubin’s rules were used to calculate results across the imputed datasets for MVPA, TPA, SB and BMI-z.

## 3. Results

**Figure 1:**
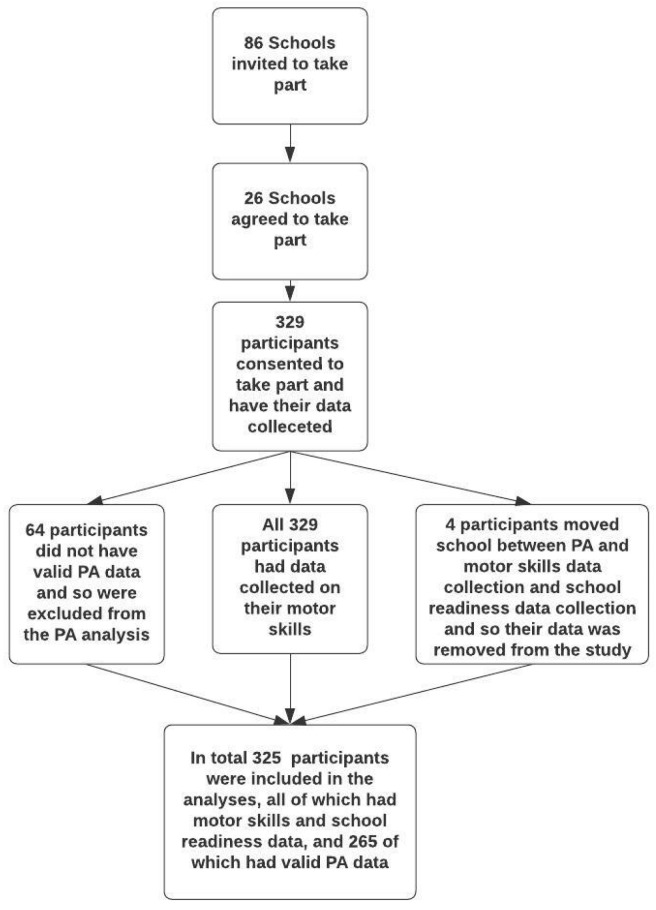
Flowchart showing data-collection and analysis procedure.

The study recruited 329 children from 26 primary schools. School-readiness data were collected for 325 of the children, as four children had moved schools between PA and motor-skill data collection and school-readiness data collection. All 329 children had motor skills and PA data collected. However, 64 children did not have valid accelerometer data for their PA data to be included in the PA analyses. Figure 1 shows the data-collection and analysis procedure.

### 3.1. Descriptive and Comparative Analysis

The average age at exposure (i.e., when PA and motor-skill data were collected) was 5.0 (SD = 0.4) years, while age at outcome (i.e., when school-readiness data were recorded) was 5.4 (0.3) years. The average BMI-z score of children was 0.5 (1.1), which is indicative of a healthy weight, and the average SES score correlated to living in the fifth decile of deprivation. Concerning school readiness, 79.6% (n = 258) of children achieved a good level of development, with 83.8% (n = 129) of girls and 75.9% (n = 129) of boys achieving a good level of development, although the difference was not statistically significant (*p* = 0.052).

The average total motor-skill score is in the 53rd percentile, according to MABC-2 age-standardised scores. Concerning the domains of motor skills, fine motor skill was in the 49th, object control in the 51st and balance skills in the 58th percentile. Finally, the locomotor score measured by TGMD-2 was in the 34th percentile. According to the MABC-2 motor-skill classification system, 5.2% of the sample had evidence of motor delay, 8.2% of the sample were at risk of motor delay and 86.6% of the sample were normally developing.

Table 2 shows the frequencies of children for different motor-skill classifications, based on the MABC-2, and whether they achieved a good level of development or not. It also shows the average school-readiness score for each motor-skill classification. Children who had evidence of a motor delay (35.3%) or were at risk of motor delay (66.7%) were less likely to achieve a good level of development compared to children who were normally developing (83.6%). There were significant differences in school-readiness scores for children in each motor-skill classification. Post hoc Tukey tests indicated that children with evidence of motor delay scored significantly worse on school-readiness score compared to children at risk of developmental delay (*p* = 0.02) and normally developing children (*p* = <0.01).

Statistically significant differences were noted between children who had valid PA data and those who did not, according to age at exposure (valid PA = 5.0, SD = 0.4, not valid PA = 4.9, SD = 0.3, *p* = 0.04), object-control skills (valid PA = 9.9, SD = 2.7, not valid PA = 10.9, SD = 2.6, *p* = 0.01), school-readiness score (valid PA = 26.2, SD = 5.6, not valid PA = 23.4, SD = 4.8, *p* = <0.01), and the percentage of children achieving a good level of development (valid PA = 82.2%, not valid PA = 68.9%, *p* = 0.02). Children without PA data were, on average, significantly younger, had better object-control skills but lower school-readiness score and were less likely to achieve a good level of development.

Table 3 shows the average amount of PA and motor-skill and school-readiness score for the sample, as well as comparisons between sexes. There were sex differences in motor skills, with girls scoring significantly higher for fine motor, balance, and locomotor skills compared to boys. Sex differences also existed for school readiness and physical activity; girls scored significantly higher for school-readiness score compared to boys. However, boys engaged in significantly more LPA, MVPA, VPA and TPA compared to girls.

### 3.2. Correlation Analysis

Table 4 shows the association between demographic, anthropometric, PA and motor-skill variables with achieving and not achieving a good level of development and school-readiness score. Age at exposure and age at outcome were significantly associated with achieving a good level of development and school-readiness score, with older children being more likely to achieve a good level of development and score higher for school-readiness score. BMI-z was not associated with achieving a good level of development or school-readiness score. There was no significant difference between children who did and did not achieve a good level of development, according to SES. However, SES was associated with school-readiness score, with children from more affluent areas achieving higher school-readiness scores. Likewise, SB was not associated with a good level of development, although it was associated with school-readiness score. Children who engaged in more SB were more likely to score higher in school-readiness score. LPA, MVPA, VPA and TPA were not associated with a good level of development or school-readiness score. Children who achieved a good level of development had higher motor-skill scores for all motor-skill domains compared to children who did not achieve a good level of development. All domains of motor skills, except object-control skills, were associated with school-readiness score, as children with better motor skills scored higher.

### 3.3. Regression Analysis

Using a complete case analysis, logistic regression models (Table 5) revealed that neither SB (*p* = 0.36), MVPA (*p* = 0.23) or TPA (*p* = 0.83) were significant predictors of achieving or not achieving a good level of development when the analysis was adjusted for sex, age at exposure, BMI-z and SES. However, all motor-skill variables were significant predictors of a child achieving a good level of development when adjusted for the same variables. Total motor skills were the most significant predictor, with a one-unit increase in total motor-skill score resulting in the odds of achieving a good level of development increasing by 31%. A one unit increase in other motor skills domains resulted in the odds of achieving a good level of development increasing by 26% for fine motor skills, 17% for balance, 14% for object control skills and 12% for locomotor skills. The total motor-skills model also predicted more of the variance in a good level of development (18.6%) (Nagelkerke R^2^) than any other model (fine motor 16.7%, locomotor 15.2%, object control 10.5%, balance 8.1%).

Due to missing PA data, sensitivity analyses were conducted using imputed data. Results with the imputed data were similar for complete case data, showing that neither MVPA (OR = 1.01, *p* = 0.16), TPA (OR = 1.00, *p* = 0.77) or SB (OR = 1.00, *p* = 0.35) were significant predictors of a good level of development. A separate analysis was conducted to explore the odds ratio (OR) of achieving a good level of development depending on motor-skill classification. Children with evidence of a motor delay (total motor skills = 5) had a lower OR of achieving a good level of development (OR = 1.7) compared with children at risk of motor delay (total motor skills = 5) (OR =1.9) or children who were normally developing (total motor skills = 8) (OR = 2.0).

Linear regression models adjusted for sex, age at exposure, BMI-z and SES (Table 6) found that MVPA (*p* = 0.45) and TPA (*p* = 0.11) did not significantly predict school-readiness score. However, SB significantly predicted school-readiness score, with the SB model accounting for 11.7% of the variance in school-readiness score. Furthermore, all motor-skill variables, except object-control skills, were significant predictors of school-readiness score. In particular, the results show that a one-unit increase in total motor skills led to a 0.56 increase in school-readiness score; this increase was higher compared to any other model (fine motor 0.54, balance 0.36, object control 0.22, locomotor 0.20). The fine motor skills model predicted more of the variance in school-readiness score (17.9%) (R^2^) compared to any other model (total motor 17.6%, balance 14.0%, locomotor 13.1%, object-control skills 10.8%).

Due to missing PA data, sensitivity analyses were conducted using imputed data. Multiple imputation sensitivity analyses found similar results to the complete case analyses. The results showed that MVPA (*B* = −0.01, *p* = 0.63) and TPA (*B* = 0.00, *p* = 0.13) were not significant predictors of school-readiness score. However, SB remained a significant predictor (*B* = 0.02, *p* = 0.02).

## 4. Discussion

The aim of this study was to examine the association between school readiness, recorded as a good level of development and school-readiness score, using the EYFSP, with PA, SB and motor skills. The study found that all motor-skill variables predicted a good level of development, with a one-unit increase in total motor-skill score resulting in a 31% increase in the odds of achieving a good level of development. Likewise, all motor-skill domains, except object-control skills, were significant predictors of school-readiness score, with higher scores for total motor, fine motor, balance, and locomotor skills predicting higher school-readiness scores. Furthermore, the study found that children with developmental delay or at risk of developmental delay, according to the MABC-2, were less likely to achieve a good level of development and more likely to have lower school-readiness scores than normally developing children. In terms of PA and SB, neither MVPA nor TPA were significantly predictive of school-readiness score or a good level of development. However, SB was significantly predictive of school-readiness score.

These findings build on previous research looking at different areas of school readiness and motor skills [23,26,44]. Several studies have demonstrated the predictive value of fine motor skills for academic achievement in terms of maths and literacy from preschool to later school years [23,45]. Gross motor skills have also been associated with academic outcomes [19]. A combined movement and storytelling intervention in preschool children found that language ability was significantly improved in the intervention compared to movement or storytelling interventions alone [19].

The finding that motor skills are strongly associated with school readiness in this study is perhaps unsurprising, given the exemplification materials for several early learning goals. For example, the physical development area of learning specifically describes the ability to control and coordinate both large and small movements, as well as the ability to dress and go to the toilet independently, which requires gross motor skills, such as maintaining balance, reaching in all directions, and crossing the midline. Furthermore, both gross (hop confidently and skip in time to music; dress and undress independently) and fine motor skills (holding paper in position, correct pencil grip, writing on lines and controlling letter size, successfully fastening buttons or laces) are explicitly described within the exceeding category for each early learning goal [4].

The lack of association between TPA and MVPA and school readiness should be viewed with caution since children who did not have PA data were less likely to achieve school readiness. However, it is important to note that the physical development area of learning only requires a child to know the importance of physical exercise but does not state that the children need to demonstrate engagement in large amounts of PA. Instead, it focusses on the control, coordination and quality of motor skills, such as hopping and skipping [4]. This finding is supported by a recent systematic review [46], which found no clear consensus on the role of PA for academic outcomes in early childhood. Furthermore, it may also be possible that children who engage in higher levels of PA do less sedentary activities, which may be perceived as better for the development of school readiness, such as reading, writing, fine motor activities, socialising and communicating. This is supported by the finding that SB was a significant predictor of school-readiness score, although higher SB did not significantly increase the odds ratio of achieving a good level of development. This suggests that a good level of development can be achieved across a range of time spent in SB, but to achieve high school-readiness scores, more time in SB is required. Whilst the context of children’s SB was not explored in this study, Marr et al. (2003) found that children spent 46% of their time in preschool on fine motor activities [47], which are likely to be conducted whilst sedentary. Other activities that would increase the likelihood of children being school-ready, such as mathematics, literacy and storytelling, also tend to be sedentary tasks [4]. Future studies should explore the context of early years children’s SB and its association with school readiness in order to understand the type of sedentary behaviours that are most beneficial. Therefore, interventions to improve school readiness may focus on encouraging children to spend time in activities that, whilst sedentary, improve the likelihood of being school-ready, such as fine motor skills, reading and writing, whilst reducing sedentary behaviours that have negative health outcomes, such as excess, poor-quality screen time [48].

### 4.1. Strengths and Limitations

The main strength of this study is that it provides novel evidence of school readiness, assessed by the UK EYFSP and with accelerometer-assessed PA and motor-skill assessments, which have been validated in this age group [37,38]. Furthermore, the large sample size allowed a robust statistical analysis, including multiple imputation, and offered a more accurate and comprehensive evaluation of results.

The main limitation of the study is the missing PA data and the evidence that data were missing not at random. Physical-activity data were missing for children who had significantly higher object-control skills, lower school-readiness scores, and a lower percentage of children achieving a good level of development. This might have skewed the data in the direction of no association between PA and school readiness. However, multiple imputation sensitivity analyses were conducted to mediate the effect of missing data, and these analyses consistently demonstrated similar findings to that of the complete case analyses.

Furthermore, the sampling strategy may have attracted participants with parents who promote their PA, motor-skill development and school readiness beyond the typical parent, potentially making the sample unrepresentative. This may explain why MVPA in this study (99.6 ± 23.6 min/day) was higher than previously reported [49,50] and a larger percentage of children in the sample achieved school readiness than the national average (79.6% in this sample compared to 71.8% in England) [7]. Another limitation of this study is the use of accelerometers to assess PA, which does not accurately assess activities such as cycling and is removed for water-based activities, so may underestimate habitual PA levels. Another data-collection-related limitation is the fact that a mixture of processes and product assessments were used to assess motor skills. However, these data were presented separately in this study. A further limitation to the study is the disparity between the time the exposure variables (PA and motor skills) were measured and the time the outcome (school readiness) was measured for different participants. Children who had their PA assessed closer to the school-readiness data-collection date would provide a more accurate description of the association between school readiness and PA, particularly given the speed at which children develop in early years [51]. However, to attenuate this effect, all statistical analyses were adjusted for age at exposure.

### 4.2. Recommendations for Policy and Practice

The main finding of this study was the positive association between motor skills and school readiness. The promotion of gross and fine motor skills in schools may increase the number of children who achieve school readiness, who will then be more likely to achieve better educational outcomes [3,5,52,53]. Furthermore, by increasing the number of children who achieve school readiness, children are better able to access the learning delivered in formal schooling. This ultimately means that teachers can dedicate less time to developing the skills of children who are not school-ready and allocate more time to delivering the core curriculum, which would confer benefits for all children in the class.

The strong association presented in this study between motor skills and schools readiness demonstrates that policymakers and commissioners should support schools to implement interventions that promote the acquisition of motor skills ss they may increase the likelihood of a child achieving school readiness, as well as provide a multitude of health benefits. Firstly, as motor skills are developed in sequence, gross motor skill development could be prioritised as this may lead to improvements in fine motor skills [54]. Furthermore, better motor skills may lead to increased engagement in PA in early years and beyond [13,55].

## 5. Conclusions

This study expands on the well documented benefits of PA and motor skills for early years [13,56,57,58]. We found that early-years motor-skill ability significantly predicts children’s achievement of school readiness. However, the study also shows that children’s early-years PA levels do not predict school readiness, although SB is a positive predictor. This study provides valuable evidence for strategies to improve early-years development and future outcomes. The promotion of motor skills in parallel with developmentally positive sedentary behaviours, whilst maintaining sufficient PA, in early years may help to increase the number of children achieving school readiness and may lead to long-term benefits in educational, social and physical development.

## Figures and Tables

**Table 1:** EYFSP areas of learning and early learning goals [4].

Areas of Learning	Early Learning Goals
Prime Areas of Learning	
Communication and Language	Listening and attention
	Understanding
	Speaking
Physical Development	Moving and handling
	Health and self-care
Personal, Social and Emotional Development	Making relationships
	Self-confidence and self-awareness
	Managing feelings and behaviour
Specific Areas of Learning	
Literacy	Reading
	Writing
Mathematics	Numbers
	Shape, space and measure

**Table 2:** Frequency of children achieving a good level of development and mean school-readiness score for each motor-skill classification.

Motor-Skill Classification	Good Level of Development		School-Readiness Score (SD)
	Achieved a Good Level of Development (%)	Not Achieved a Good Level of Development (%)	
Evidence of Motor Delay	6 (35.3)	11 (64.7)	19.3 (4.9)
Risk of Motor Delay	18 (66.7)	9 (33.3)	23.8 (6.4)
Normally Developing	235 (83.6)	46 (16.4)	26.3 (5.2)

Evidence of motor delay = total MABC-2 score ≤5, risk of motor delay = total MABC-2 score 6–7, normally developing = total MABC-2 score ≥8.

**Table 3:** Average and sex differences for school-readiness score, activity levels and motor-skill scores.

	Total	SD	Boys (n = 170)	SD	Girls (n = 155)	SD	Sex Differences (*p*)
School-Readiness Score	25.7	5.6	24.9	5.1	26.5	5.9	0.01
Physical Activity			(n = 137)		(n = 128)		
SB (min/day)	261.6	45.4	256.2	46.7	267.3	43.5	0.05
LPA (min/day)	271.6	34.0	276.1	36.9	266.8	30.1	0.03
MVPA (min/day)	99.6	23.6	104.1	24.1	94.8	22.2	<0.01
VPA (min/day)	29.0	12.1	30.7	13.3	27.2	10.6	0.02
TPA (cpm)	771.7	154.4	791.6	162.9	750.4	142.3	0.03
Wear Time (min/day)	634.1	53.6	636.4	54.5	631.7	52.8	0.47
Motor Skills			(n = 170)		(n = 155)		
Total Motor Skills (1–18)	10.3	2.9	10.0	2.9	10.6	2.8	0.07
Fine Motor (1–18)	9.8	3.1	9.5	3.2	10.2	3.0	0.04
Object Control (1–18)	9.9	2.7	10.1	2.9	9.6	2.4	0.14
Balance (1–18)	11.1	3.2	10.5	3.1	11.7	3.2	<0.01
Locomotor (0–48)	25.9	6.6	25.2	6.7	26.7	5.9	0.03

SB = sedentary behaviour, LPA = light physical activity, MVPA = moderate to vigorous physical activity. VPA = vigorous physical activity, TPA = total physical activity.

**Table 4:** Association between demographic, anthropometric and physical activity variables and a good level of development and school-readiness score.

	Good Level of Development			School-Readiness Score (*r*)
	Achieved a Good Level of Development Mean (SD)	Not Achieved a Good Level of Development Mean (SD)	Mean Difference	
Anthropometric/Demographic				
Age at exposure (years)	5.0 (0.3)	4.8 (0.4)	0.2 **	0.24 **
Age at outcome (years)	5.5 (0.3)	5.3 (0.3)	0.2 **	0.26 **
BMI-z	0.5 (1.1)	0.4 (1.2)	0.1	−0.02
SES	15,487.8 (10,239.7)	14,950.7 (10,213.9)	537.1	0.12 *
Physical Activity				
SB min/day	263.2 (44.6)	253.1 (48.5)	10.1	0.18 **
LPA min/day	271.7 (33.3)	270.6 (35.5)	1.1	−0.02
MVPA min/day	100.1 (23.4)	96.2 (24.2)	3.9	−0.07
VPA min/day	29.5 (12.3)	26.2 (11.2)	−3.3	−0.01
TPA (CPM)	770.5 (153.6)	767.1 (160.4)	−3.4	−0.12
Motor Skills				
Total motor (1–19)	10.6 (2.6)	9.0 (3.2)	1.6 **	0.26 **
Fine (1–19)	10.0 (2.9)	8.8 (3.7)	1.2 *	0.21 **
Object control (1–19)	10.3 (2.7)	9.3 (2.7)	1.0 *	0.10
Balance (1–19)	11.4 (3.2)	9.9 (3.3)	1.5 **	0.23 **
Locomotor (0–48)	27.2 (4.8)	24.0 (5.6)	3.2 **	0.23 **

* <0.05, ** <0.01. BMI-z = Body Mass Index-z, SES = socio-economic status (IMD (2019) ward score), SB = sedentary behaviour, LPA = light physical activity, MVPA = moderate to vigorous physical activity. VPA = vigorous physical activity, TPA = total physical activity.

**Table 5:** Logistic regression predicting achievement of a good level of development.

	OR	95% CI		*p*
		Lower	Upper	
MVPA	1.01	0.99	1.02	0.23
TPA	1.00	1.00	1.00	0.83
SB	1.00	1.00	1.01	0.36
Total Motor Skills	1.31	1.17	1.47	<0.01
Fine Motor Skills	1.26	1.13	1.40	<0.01
Balance Skills	1.17	1.06	1.30	<0.01
Object-Control skills	1.14	1.02	1.28	0.02
Locomotor Skills	1.12	1.08	1.19	<0.01

All analyses were adjusted for sex, age at exposure, BMI-z and SES, OR = odds ratio.

**Table 6:** Linear regression predicting school-readiness score.

	*B*	SE	β	t	*p*
MVPA	−0.01	0.02	−0.05	−0.75	0.45
TPA	0.00	0.00	−0.10	−1.62	0.11
SB	0.02	0.01	0.14	2.40	0.02
Total Motor Skills	0.56	0.10	0.29	5.47	<0.01
Fine Motor Skills	0.54	0.10	0.30	5.56	<0.01
Balance Skills	0.36	0.09	0.21	3.92	<0.01
Object-Control Skills	0.22	0.11	0.10	1.92	0.06
Locomotor Skills	0.20	0.06	0.19	3.48	<0.01

All analyses were adjusted for sex, age at exposure, BMI-z and SES, OR = odds ratio.

## Data Availability

The data presented in this study are available on request from the corresponding author.
